# An Analysis on Local Convergence of Inexact Newton-Gauss Method for Solving Singular Systems of Equations

**DOI:** 10.1155/2014/752673

**Published:** 2014-03-26

**Authors:** Fangqin Zhou

**Affiliations:** Department of Mathematics and Physics, Quzhou University, Quzhou 324000, China

## Abstract

We study the local convergence properties of inexact Newton-Gauss method for singular systems of equations. Unified estimates of radius of convergence balls for one kind of singular systems of equations with constant rank derivatives are obtained. Application to the Smale point estimate theory is provided and some important known results are extended and/or improved.

## 1. Introduction

Consider the following system of nonlinear equations:

(1) $$\begin{array}{c} f\;\left(x\right)=0, \end{array}$$

where *f* : *D* ⊂ ℝ^*n*^ → ℝ^*m*^ is a nonlinear operator with its Fréchet derivative denoted by *f*′ and *D* is open and convex. In the case when *m* = *n* and *f*′(*x*) is invertible for each *x* ∈ *D*, Newton's method is a classical numerical method to find an approximation solution for such system. There are a lot of results that improve, generalize, or extend the convergence of Newton's method for solving (1). We refer the reader to the works of Deuflhard and Heindl [1], Smale [2], Wang [3], Ferreira [4], Argyros et al. [5], and the references therein. If *x*_0_ ∈ *D* is an approximation of a zero of this system, then Newton's method can be defined by the form as follows:

(2) $$\begin{array}{c} x_{k+1}=x_{k}-f^{\prime }{\left(x_{k}\right)}^{-1}f\left(x_{k}\right),\;k=0,1,2,\ldots . \end{array}$$

When *f*′(*x*) is not invertible, we choose its Moore-Penrose inverse *f*′(*x*)^†^ instead of its classical inverse and call it Gauss-Newton's method given as follows:

(3) $$\begin{array}{c} x_{k+1}=x_{k}-f^{\prime }{\left(x_{k}\right)}^{†}f\left(x_{k}\right),\;k=0,1,2,\ldots . \end{array}$$

Let *A* : ℝ^*n*^ → ℝ^*m*^ be a linear operator (or an *m* × *n* matrix). Recall that an operator (or *n* × *m* matrix) *A* ^†^ : ℝ^*m*^ → ℝ^*n*^ is the Moore-Penrose inverse of *A*, if it satisfies the following four equations:

(4) $$\begin{array}{c} A^{†}AA^{†}=A^{†},\;\;AA^{†}A=A;\\ {\left(AA^{†}\right)}^{*}=AA^{†},\;\;{\left(A^{†}A\right)}^{*}=A^{†}A, \end{array}$$

where A* denotes the adjoint of *A*. Let *ker*⁡*A* and im  *A* denote the kernel and image of *A*, respectively. For a subspace *E* of ℝ^*n*^, we use Π_*E*_ to denote the projection onto *E*. Then, it is clear that

(5) $$\begin{array}{c} A^{†}A=\Pi_{\ker A^{⊥}},\;\;AA^{†}=\Pi_{\text{im}A}. \end{array}$$

In particular, in the case when *A* is full row rank (or, equivalently, when *A* is surjective), *AA* ^†^ = *I*_ℝ^*m*^_; when *A* is full column rank (or equivalently, when *A* is injective), *A* ^†^ *A* = *I*_ℝ^*n*^_.

One of the disadvantages for Newton's method (2) is that it requires solving exactly the following linear equation at each step:

(6) $$\begin{array}{c} f^{\prime }\left(x_{k}\right)\left(x_{k+1}-x_{k}\right)=-f\left(x_{k}\right). \end{array}$$

To overcome this disadvantage, Dembo et al. presented in [6] the following iterative processes called inexact Newton method (*x*_0_ is an initial guess):

(7) xk+1=xk+sk,  f′(xk)sk=−f(xk)+rk, k=0,1,2,…,

where the residual control *r*_*k*_ satisfies

(8) $$\begin{array}{c} ||r_{k}||\leq \lambda_{k}||f\left(x_{k}\right)||,\;k=0,1,2,\ldots , \end{array}$$

and {*λ*_*k*_} is a sequence of forcing terms such that 0 ≤ *λ*_*k*_ < 1. In [6], it was shown that if *λ*_*k*_ ≤ *λ* < 1, then there exists *r* > 0 such that, for any initial guess *x*_0_ ∈ *B*(*ζ*, *r*), the sequence {*x*_*k*_} is well defined and converges to a solution *ζ*. Moreover, the rate of convergence of {*x*_*k*_} to *ζ* is characterized by the rate of convergence of {*λ*_*k*_} to 0.

Note that it is clear that the residual control (8) is not affine invariant (see [1] for more details about the affine invariant). To this end, Ypma used in [7] the affine invariant condition of residual control in the form

(9) $$\begin{array}{c} ||f^{\prime }{\left(x_{k}\right)}^{-1}r_{k}||\leq \lambda_{k}||f^{\prime }{\left(x_{k}\right)}^{-1}f\left(x_{k}\right)||,\;k=0,1,2,\ldots , \end{array}$$

to study the local convergence of inexact Newton method (7). And the radius of convergent result is also obtained.

To study the local convergence of inexact Newton method and inexact Newton-like method (called inexact methods for short below), Morini presented in [8] the following variation for the residual controls:

(10) $$\begin{array}{c} ||P_{k}r_{k}||\leq \lambda_{k}||P_{k}f\left(x_{k}\right)||,\;k=0,1,2,\ldots , \end{array}$$

where {*P*_*k*_} is a sequence of invertible operator from ℝ^*n*^ to ℝ^*n*^ and {*λ*_*k*_} is the forcing term. If *P*_*k*_ = *I* and *P*_*k*_ = *f*′(*x*_*k*_) for each *k*, (10) reduces to (8) and (9), respectively. Both proposed inexact methods are linearly convergent under Lipschitz condition. It is worth noting that the residual controls (10) are used in iterative methods if preconditioning is applied and lead to a relaxation on the forcing terms. But we also note that the results obtained in [8] cannot make us clearly see how big the radius of the convergence ball is. To this end, Chen and Li [9] obtained the local convergence properties of inexact methods for (1) under weak Lipschitz condition, which was first introduced by Wang in [10] to study the local convergence behavior of Newton's method (2). The results in [9] easily provide an estimate of convergence ball for the inexact methods. Furthermore, Ferreira and Gonçalves presented in [11] a new local convergence analysis for inexact Newton-like under so-called majorant condition, which is equivalent to the preceding weak Lipschitz condition.

Under the assumption that the derivative of the operator satisfies the Hölder condition, the radius of convergence ball of the inexact Newton-like methods with a new type of residual control is estimated by Li and Shen [12]. And a superlinear convergence property is proved, which extends the corresponding result in [8]. In addition, as an application of the local convergence result, they presented a slight modification of the inexact Newton-like method of [13] for solving inverse eigenvalue problems and showed that it can be regarded equivalently as one of the inexact methods considered in [12].

Recent attentions are focused on the study of finding zeros of singular nonlinear systems by Gauss-Newton's method (3). For example, Shub and Smale extended in [14] the Smale point estimate theory (including *α*-theory and *γ*-theory) to Gauss-Newton's methods for underdetermined analytic systems with surjective derivatives. For overdetermined systems, Dedieu and Shub studied in [15] the local linear convergence properties of Gauss-Newton's for analytic systems with injective derivatives and provided estimates of the radius of convergence balls for Gauss-Newton's method. Dedieu and Kim in [16] generalized both the results of the underdetermined case and the overdetermined case to such case where *f*′(*x*) is of constant rank (not necessarily full rank), which has been improved by Xu and Li in [17, 18], Ferreira et al. in [19], Argyros and Hilout in [20], and Gonçalves and Oliveira in [21].

In the last years, some authors have studied the convergence behaviour of inexact versions of Gauss-Newton's method for singular nonlinear systems. For example, Chen [22] employed the ideas of [9] to study the local convergence properties of several inexact Gauss-Newton type methods where a scaled relative residual control is performed at each iteration under weak Lipschitz conditions. Ferreira et al. presented in their recent paper [23] a local convergence analysis of an inexact version of Gauss-Newton's method for solving nonlinear least squares problems. Moreover, the radii of the convergence balls under the corresponding conditions were estimated in these two papers.

In the present paper, we study the local convergence of inexact Newton-Gauss method for the singular systems with constant rank derivatives under the hypotheses that the derivatives satisfy Lipschitz conditions with *L* average and the residual satisfies several control conditions. Unified estimates for the radius of convergence balls of inexact Newton-Gauss method are obtained. As an application to Smale approximate zeros, we obtain a gamma-type theorem which gives an estimate of the size of convergence ball of inexact Newton-Gauss method about a zero.

The rest of this paper is organized as follows. In Section 2, we introduce some preliminary notions and properties of the majorizing function. The main results about the local convergence are stated in Section 3. And finally, in Section 4, we prove the local convergence results given in Section 3.

## 2. Preliminaries

For *x* ∈ ℝ^*n*^ and a positive number *r*, throughout the whole paper, we use *B*(*x*, *r*) to stand for the open ball with radius *r* and center *x* and let $\bar{B(x,r)}$ denote its closure.

Throughout this paper, we assume that *L* is a positive nondecreasing function on [0, *R*), where *R* ∈ ℝ^+^. Let 0 ≤ *λ*, *θ* < 1 with 0 ≤ *λ* + *θ* < 1. The majorizing function *h*_*λ*,*θ*_ : [0, *R*] → ℝ corresponding to (*λ*, *θ*, *L*) is defined by

(11) $$\begin{array}{c} h_{\lambda ,\theta }\left(t\right)=-\left(1+\lambda +\theta \right)t+\overset{t}{\underset{0}{\int }}L\left(u\right)\left(t-u\right)\text{d}u,\;t\in \left[0,R\right]. \end{array}$$

Note that, in the case when *λ* = *θ* = 0, (11) reduces to

(12) $$\begin{array}{c} h_{0,0}\left(t\right)=-t+\overset{t}{\underset{0}{\int }}L\left(u\right)\left(t-u\right)\text{d}u,\;t\in \left[0,R\right). \end{array}$$

Obviously,

(13) $$\begin{array}{c} h_{\lambda ,\theta }^{\prime }\left(t\right)=-\left(1+\lambda +\theta \right)+\overset{t}{\underset{0}{\int }}L\left(u\right)\text{d}u,\;t\in \left[0,R\right),\\ h_{\lambda ,\theta }^{\prime \prime }\left(t\right)=L\left(t\right)\;\text{for}\;\;\text{a}.\text{e}.\;\;0\leq t<R. \end{array}$$

Moreover, we have *h*_*λ*,*θ*_(0) = 0, *h*_*λ*,*θ*_′(0) = −(1 + *λ* + *θ*) and *h*_*λ*,*θ*_′ is convex and strictly increasing. Set

(14)  ξ≔sup⁡⁡{t∈[0,R):h0,0′(t)<0},

(15)  ρ≔sup⁡⁡{t∈[0,ξ):hλ,θ(t)h0,0′(t)−t<t},

(16)  σ≔sup⁡⁡{t∈[0,R):B(ζ,t)⊂D}.

For the convergence analysis, we need the following useful lemma about elementary convex analysis.

**Lemma 1 (see [4])**

Let *R* > 0. If *g* : [0, *R*] → ℝ is continuously differentiable and convex, then

- (*g*(*t*) − *g*(*τt*))/*t* ≤ (1 − *τ*)*g*′(*t*), for all *t* ∈ (0, *R*) and *τ* ∈ [0,1],
- (*g*(*u*) − *g*(*τu*))/*u* ≤ (*g*(*v*) − *g*(*τv*))/*v*, for all *u*, *v* ∈ [0, *R*), *u* < *v*, and 0 ≤ *τ* ≤ 1.

The next two lemmas show that the constants *ξ* and *ρ* defined in (14) and (15), respectively, are positive.

**Lemma 2**

The constant *ξ* defined in (14) is positive and *t* − (*h*_*λ*,*θ*_(*t*)/*h*_0,0_′(*t*)) < 0, for all *t* ∈ (0, *ξ*).

**Proof**

Since *h*_0,0_′(0) = −1, there exists *δ* > 0 such that *h*_0,0_′(*t*) < 0 for all *t* ∈ (0, *δ*). Then, we get *ξ* ≥ *δ*(>0). Because *h*_*λ*,*θ*_′ is strictly increasing, *h*_*λ*,*θ*_ is strictly convex. It follows from Lemma 1(i) that

(17) $$\begin{array}{c} \frac{h_{\lambda ,\theta }\left(t\right)-h_{\lambda ,\theta }\left(0\right)}{t}\leq h_{\lambda ,\theta }^{\prime }\left(t\right)\leq h_{0,0}^{\prime }\left(t\right),\;t\in \left(0,R\right). \end{array}$$

Note that *h*_*λ*,*θ*_(0) = 0 and *h*_0,0_′(*t*) < 0, for all *t* ∈ (0, *ξ*). Thus, the inequality *t* − (*h*_*λ*,*θ*_(*t*)/*h*_0,0_′(*t*)) < 0 follows.

**Lemma 3**

The constant *ρ* defined in (15) is positive. As a consequence, |*t* − (*h*_*λ*,*θ*_(*t*)/*h*_0,0_′(*t*))| < *t*, for all *t* ∈ (0, *ρ*).

**Proof**

On one hand, by Lemma 2, it is clear that (*h*_*λ*,*θ*_(*t*)/*th*_0,0_′(*t*)) − 1 > 0, for *t* ∈ (0, *ξ*). On the other hand, we can obtain from Lemma 1(i) that

(18) $$\begin{array}{c} \underset{t\to 0}{\lim}\left(\frac{h_{\lambda ,\theta }\left(t\right)}{{th}_{0,0}^{\prime }\left(t\right)}-1\right)=0. \end{array}$$

Then, we conclude that there exists *δ* > 0 such that

(19) $$\begin{array}{c} 0<\frac{h_{\lambda ,\theta }\left(t\right)}{{th}_{0,0}^{\prime }\left(t\right)}-1<1,\;t\in \left(0,\xi \right). \end{array}$$

Therefore, *ρ* is positive.

Let

(20) $$\begin{array}{c} r:=\min\left\{\rho ,\sigma \right\}, \end{array}$$

where *ρ* and *σ* are given in (15) and (16), respectively. For any starting point *x*_0_ ∈ *B*(*ζ*, *r*)∖{*ζ*}, let {*t*_*k*_} denote the sequence generated by

(21) $$\begin{array}{c} t_{0}=||x_{0}-\zeta ||,\;t_{k+1}=\left|t_{k}-\frac{h_{\lambda ,\theta }\left(t_{k}\right)}{h_{0,0}^{\prime }\left(t_{k}\right)}\right|,\;k=0,1,2,\ldots . \end{array}$$

**Lemma 4**

The sequence {*t*_*k*_} given by (21) is well defined, is strictly decreasing, is contained in (0, *ρ*), and converges to 0.

**Proof**

Since 0 < *t*_0_ = ||*x*_0_ − *ζ*|| < *r* ≤ *ρ*, using Lemma 3, one has that {*t*_*k*_} is well defined, strictly decreasing, and contained in [0, *ρ*). Thus, there exists t* ∈ [0, *ρ*) such that lim⁡_*k*→*∞*_⁡*t*_*k*_ = t*; that is, we have

(22) $$\begin{array}{c} 0\leq t^{*}=\frac{h_{\lambda ,\theta }\left(t^{*}\right)}{h_{0,0}^{\prime }\left(t^{*}\right)}-t^{*}<\rho . \end{array}$$

If t* ≠ 0, it follows from Lemma 3 that

(23) $$\begin{array}{c} \frac{h_{\lambda ,\theta }\left(t^{*}\right)}{h_{0,0}^{\prime }\left(t^{*}\right)}-t^{*}<t^{*}. \end{array}$$

This is a contradiction. So *t*_*k*_ → 0 as *k* → *∞*. This completes the proof.

The notion of the *L*-average Lipschitz condition for semilocal convergence analysis was introduced by Li and Ng in [24], which is a modification of the one that was first introduced by Wang in [3], where the terminology of “the center Lipschitz condition in the inscribed sphere with *L* average” was used. This notion was used to study the semilocal convergence of Newton's method (2) to solve singular systems of equation with constant rank derivatives by Xu and Li in [18] and Li et al. in [25]. As for the local convergence analysis, we can also introduce the similar definition.

**Definition 5**

Let *r* > 0 be such that *B*(*ζ*, *r*) ⊂ *D*. Then, *f*′ is said to satisfy the *L*-average Lipschitz condition on *B*(*ζ*, *r*) if

(24) $$\begin{array}{c} ||f^{\prime }{\left(\zeta \right)}^{†}\left[f^{\prime }\left(x\right)-f^{\prime }\left(\zeta +\tau \left(x-\zeta \right)\right)\right]||\leq \overset{||x-\zeta ||}{\underset{\tau ||x-\zeta ||}{\int }}L\left(u\right)\text{d}u, \end{array}$$

for any *x* ∈ *B*(*ζ*, *r*) and *τ* ∈ [0,1].

This definition is a modification of the one in [10], where the terminology of “the radius Lipschitz condition with the *L* average” was used. In the case when *f*′(*ζ*) is not surjective (see [15, 16]), the information on im*f*′(*ζ*)^⊥^ may be lost. To this end, we need to modify the above notion to suit the case when *f*′(*ζ*) is not surjective.

**Definition 6**

Let *r* > 0 be such that *B*(*ζ*, *r*) ⊂ *D*. Then, *f*′ is said to satisfy the modified *L*-average Lipschitz condition on *B*(*ζ*, *r*) if

(25) $$\begin{array}{c} ||f^{\prime }{\left(\zeta \right)}^{†}||||f^{\prime }\left(x\right)-f^{\prime }\left(\zeta +\tau \left(x-\zeta \right)\right)||\leq \overset{||x-\zeta ||}{\underset{\tau ||x-\zeta ||}{\int }}L\left(u\right)\text{d}u, \end{array}$$

for any *x* ∈ *B*(*ζ*, *r*) and *τ* ∈ [0,1].

The notion of the *γ*-condition for operators in Banach spaces was introduced in [26] by Wang and Han to study the Smale point estimate theory.  Definition 7 about *γ*-condition and the related Lemma 8 are taken from [25].

**Definition 7 (see [25])**

Suppose that *γ* > 0 and *f* has continuous second derivative. Let 0 < *r* ≤ 1/*γ* be such that *B*(*ζ*, *r*) ⊂ *D*. *f* is said to satisfy the *γ*-condition (resp., the modified *γ*-condition) on *B*(*ζ*, *r*) if (26) (resp., (27)) holds as follows:

(26) $$\begin{array}{c} ||f^{\prime }{\left(\zeta \right)}^{†}f^{\prime \prime }\left(x\right)||\leq \frac{2\gamma }{{\left(1-\gamma ||x-\zeta ||\right)}^{3}}\;\text{for}\;\;\text{each}\;\;x\in B\left(\zeta ,r\right), \end{array}$$

(27) $$\begin{array}{c} ||f^{\prime }{\left(\zeta \right)}^{†}||||f^{\prime \prime }\left(x\right)||\leq \frac{2\gamma }{{\left(1-\gamma ||x-\zeta ||\right)}^{3}}\;\text{for}\;\;\text{each}\;\;x\in B\left(\zeta ,r\right). \end{array}$$

**Lemma 8 (see [25])**

Suppose that *γ* > 0 and *f* has continuous second derivative. Let 0 < *r* ≤ 1/*γ* be such that *B*(*ζ*, *r*) ⊂ *D*. Then, *f* satisfies the *γ*-condition (resp., the modified *γ*-condition) on *B*(*ζ*, *r*) if and only if *f*′ satisfies the *L*-average Lipschitz condition (resp., the modified *L*-average Lipschitz condition) on *B*(*ζ*, *r*) with *L*(*u*) = 2*γ*/(1 − *γu*)^3^, *u* ∈ [0,1/*γ*).

## 3. Local Convergence for Inexact Newton-Gauss Method

In this section, we state our main results of local convergence for inexact Newton-Gauss method (7). Recall that the system (1) is a surjective-underdetermined (resp., injective-overdetermined) system if the number of equations is less (resp., greater) than the number of unknowns and *f*′(*x*) is of full rank for each *x* ∈ *D*. Note that, for surjective-underdetermined systems, the fixed points of the Newton operator *N*_*f*_(*x*): = *x* − *f*′(*x*)^†^ *f*(*x*) are the zeros of *f*, while, for injective-overdetermined systems, the fixed points of *N*_*f*_ are the least square solutions of *f*(*x*) = 0, which, in general, are not necessarily the zeros of *f*.

Our first result concerned the local convergence properties of inexact Newton-Gauss method for general singular systems with constant rank derivatives.

**Theorem 9**

Let *f* : *D* ⊂ ℝ^*n*^ → ℝ^*m*^ be continuously Fréchet differentiable nonlinear operator, and *D* is open and convex. Suppose that *f*(*ζ*) = 0, *f*′(*ζ*) ≠ 0 and that *f*′ satisfies the modified *L*-average Lipschitz condition (25) on *B*(*ζ*, *r*), where *r* is given in (20). In addition, one assumes that rank⁡*f*′(*x*) ≤ rank⁡*f*′(*ζ*), for any *x* ∈ *B*(*ζ*, *r*), and that

(28) $$\begin{array}{c} ||\left[I_{{\mathbb{R}}^{n}}-f^{\prime }{\left(x\right)}^{†}f^{\prime }\left(x\right)\right]\left(x-\zeta \right)||\leq \theta ||x-\zeta ||,\;x\in B\left(\zeta ,r\right), \end{array}$$

where the constant *θ* satisfies 0 ≤ *θ* < 1. Let {*x*_*k*_} be sequence generated by inexact Newton-Gauss method with any initial point *x*_0_ ∈ *B*(*ζ*, *r*)∖{*ζ*} and the conditions for the residual *r*_*k*_ and the forcing term *λ*_*k*_:

(29) ||rk||≤λk||f(xk)||, 0≤λkκ(f′(xk))≤λ,                k=0,1,2,…,

where *κ*(*A*): = ||*A* ^†^||||*A*|| denotes the condition number of *A* ∈ ℝ^*m*×*n*^. Then, {*x*_*k*_} converges to a zero *ζ* of *f*′(·)^†^ *f*(·) in $\bar{B(\zeta ,r)}$. Moreover, one has the following estimate:

(30) $$\begin{array}{c} ||x_{k+1}-\zeta ||\leq \frac{t_{k+1}}{t_{k}}||x_{k}-\zeta ||,\;k=0,1,2,\ldots , \end{array}$$

where the sequence {*t*_*k*_} is defined by (21).

**Remark 10**

If taking *λ* = 0 (in this case, *λ*_*k*_ = 0 and *r*_*k*_ = 0) in Theorem 9, we obtain the local convergence of Newton's method for solving the singular systems, which has been studied by Dedieu and Kim in [16] for analytic singular systems with constant rank derivatives and Li et al. in [25] for some special singular systems with constant rank derivatives. Now, we obtain that the convergence ball *r* satisfies

(31) $$\begin{array}{c} \frac{\int_{0}^{r}L\left(u\right)u\;\text{d}u}{r\left(\left(1-\theta \right)-\int_{0}^{r}L\left(u\right)\text{d}u\right)}\leq 1,\;\theta \in \left[0,1\right). \end{array}$$

If *f*′(*x*) is full column rank for every *x* ∈ *B*(*ζ*, *r*), then we have *f*′(*x*)^†^ *f*′(*x*) = *I*_ℝ^*n*^_. Thus,

(32) $$\begin{array}{c} ||\left[I_{{\mathbb{R}}^{n}}-f^{\prime }{\left(x\right)}^{†}f^{\prime }\left(x\right)\right]\left(x-\zeta \right)||=0; \end{array}$$

that is, *θ* = 0. We immediately have the following corollary.

**Corollary 11**

Suppose that rank⁡*f*′(*x*) ≤ rank⁡*f*′(*ζ*) and that

(33) $$\begin{array}{c} ||\left[I_{{\mathbb{R}}^{n}}-f^{\prime }{\left(x\right)}^{†}f^{\prime }\left(x\right)\right]\left(x-\zeta \right)||=0, \end{array}$$

for any *x* ∈ *B*(*ζ*, *r*). Suppose that *f*(*ζ*) = 0, *f*′(*ζ*) ≠ 0 and that *f*′ satisfies the modified *L*-average Lipschitz condition (25). Let {*x*_*k*_} be sequence generated by inexact Newton-Gauss method with any initial point *x*_0_ ∈ *B*(*ζ*, *r*)∖{*ζ*} and the condition (29) for the residual *r*_*k*_ and the forcing term *λ*_*k*_. Then, {*x*_*k*_} converges to a zero *ζ* of *f*′(·)^†^ *f*(·) in $\bar{B(\zeta ,r)}$. Moreover, one has the following estimate:

(34) $$\begin{array}{c} ||x_{k+1}-\zeta ||\leq \frac{t_{k+1}}{t_{k}}||x_{k}-\zeta ||,\;k=0,1,2,\ldots , \end{array}$$

where the sequence {*t*_*k*_} is defined by (21) for *θ* = 0.

In the case when *f*′(*ζ*) is full row rank, the modified *L*-average Lipschitz condition (25) can be replaced by the *L*-average Lipschitz condition (24).

**Theorem 12**

Suppose that *f*(*ζ*) = 0, *f*′(*ζ*) is full row rank, and *f*′ satisfies the *L*-average Lipschitz condition (24) on *B*(*ζ*, *r*), where *r* is given in (20). In addition, one assumes that rank⁡*f*′(*x*) ≤ rank⁡*f*′(*ζ*) for any *x* ∈ *B*(*ζ*, *r*) and that condition (28) holds. Let {*x*_*k*_} be sequence generated by inexact Newton-Gauss method with any initial point *x*_0_ ∈ *B*(*ζ*, *r*)∖{*ζ*} and the conditions for the residual *r*_*k*_ and the forcing term *λ*_*k*_:

(35) $$\begin{array}{c} ||f^{\prime }{\left(\zeta \right)}^{†}r_{k}||\leq \lambda_{k}||f^{\prime }{\left(\zeta \right)}^{†}f\left(x_{k}\right)||,\\ 0\leq \lambda_{k}\kappa \left(f^{\prime }{\left(\zeta \right)}^{†}f^{\prime }\left(x_{k}\right)\right)\leq \lambda ,\;k=0,1,2,\ldots . \end{array}$$

Then, {*x*_*k*_} converges to a zero *ζ* of *f*(·) in $\bar{B(\zeta ,r)}$. Moreover, one has the following estimate:

(36) $$\begin{array}{c} ||x_{k+1}-\zeta ||\leq \frac{t_{k+1}}{t_{k}}||x_{k}-\zeta ||,\;k=0,1,2,\ldots , \end{array}$$

where the sequence {*t*_*k*_} is defined by (21).

**Theorem 13**

Suppose that *f*(*ζ*) = 0, *f*′(*ζ*) is full row rank, and *f*′ satisfies the* L*-average Lipschitz condition (24) on *B*(*ζ*, *r*), where *r* is given in (20). In addition, one assumes that rank⁡*f*′(*x*) ≤ rank⁡*f*′(*ζ*) for any *x* ∈ *B*(*ζ*, *r*) and that condition (28) holds. Let {*x*_*k*_} be sequence generated by inexact Newton-Gauss method with any initial point *x*_0_ ∈ *B*(*ζ*, *r*)∖{*ζ*} and the conditions for the control residual *r*_*k*_ and the forcing term *λ*_*k*_:

(37) ||f′(xk)†rk||≤λk||f′(xk)†f(xk)||, 0≤λkκ(f′(xk))≤λ,k=0,1,2,….

Then, {*x*_*k*_} converges to a zero *ζ* of *f*(·) in $\bar{B(\zeta ,r)}$. Moreover, one has the following estimate:

(38) $$\begin{array}{c} ||x_{k+1}-\zeta ||\leq \frac{t_{k+1}}{t_{k}}||x_{k}-\zeta ||,\;k=0,1,2,\ldots , \end{array}$$

where the sequence {*t*_*k*_} is defined by (21).

**Remark 14**

In the case when *f*′(*ζ*) is invertible in Theorem 13, we obtain the local convergence results of inexact Newton-Gauss method for nonsingular systems, and the convergence ball *r* in this case satisfies

(39) $$\begin{array}{c} \frac{\int_{0}^{r}L\left(u\right)u\;\text{d}u}{r\left(\left(1-\lambda \right)-\int_{0}^{r}L\left(u\right)\text{d}u\right)}\leq 1,\;\lambda \in \left[0,1\right). \end{array}$$

In particular, if taking *λ* = 0, the convergence ball *r* determined in (39) reduces to the one given in [10] by Wang and the value *r* is the optimal radius of the convergence ball when the equality holds. Then, we can conclude that vanishing residuals, Theorem 13 merges into the theory of Newton's method.

The result below is an extension of the Smale approximate zeros. We first recall the notion of the approximate zero of an analytic operator *f* from the domain *D* in a Banach space to another. In [2], Smale proposed two kinds of the notion: the first kind (in sense of ||*x*_*k*_ − *x*_*k*−1_||) and the second kind (in sense of ||*x*_*k*_ − *ζ*||) of an approximate zero. A more reasonable definition for the second kind was presented in [27]; see also [28]. The notion of the approximate zero in the sense of ||*f*′(*x*_0_)^−1^ *f*(*x*_*k*_)|| was defined in [29], which is equivalent to the first kind (see [3]). The following unified definition is taken from [3].

**Definition 15 (see [3])**

Let *x*_0_ ∈ *D* be such that the sequence {*x*_*k*_} generated by Newton's method (2) is well defined and satisfies

(40) $$\begin{array}{c} e\left(x_{k}\right)\leq {\left(\frac{1}{2}\right)}^{2^{k-1}}e\left(x_{k-1}\right),\;k=1,2,\ldots , \end{array}$$

where *e*(*x*_*k*_) denotes some measurement of the approximation degree between *x*_*k*_ and the zero point *ζ*. Then, *x*_0_ is called an approximate zero of *f* in sense of *e*(*x*_*k*_).

The concepts of an approximate zero for Gauss-Newton method (3) for solving singular systems of equations and inexact Newton method (7) for solving nonsingular systems of equations are proposed in [25, 30], respectively. We now extend the notion of approximate zeros to inexact Newton-Gauss method for solving singular systems of equations.

**Definition 16**

Let *x*_0_ ∈ *D* be such that the sequence {*x*_*k*_} generated by inexact Newton-Gauss method (7) converges to a zero *ζ* of *f*′(·)^†^ *f*(·) (resp., *f*) and satisfies (40). Then, *x*_0_ is called an INM-approximate solution (resp., approximate zero) of *f* in sense of *e*(*x*_*k*_).

For the remainder of this subsection, let *L* be the function defined by

(41) $$\begin{array}{c} L\left(u\right)=\frac{2\gamma }{{\left(1-\gamma u\right)}^{3}}\;\text{for}\;\;\text{each}\;\;u\;\;\text{with}\;\;0\leq u<\frac{1}{\gamma }. \end{array}$$

Then,

(42) hλ,θ(t)=−(1+λ+θ)t+γt21−γt, t∈[0,1γ),λ,θ∈[0,1)                    with  0≤λ+θ<1,

and the constants *ξ* and *ρ* defined in (14) and (15), respectively, have the following concrete forms:

(43) $$\begin{array}{c} \xi =\frac{2-\sqrt{2}}{2\gamma }, \end{array}$$

(44) ρ=((5+2(λ+θ)) −(5+2(λ+θ))2−4(1+λ+θ)(2+λ+θ))×(2(2+λ+θ)γ)−1.

To state our gamma-type theorem for inexact Newton-Gauss method (7), we introduce some more notations. Let

(45) ϕ(t)=4γ2(λ+θ)t3−2γ(3λ+4θ+γ)t2+2(λ+θ+3γ)t−1, λ,θ∈[0,1),  γ>0.

Since *ϕ*(0) = −1 and $\phi (\xi )=\left((3-2\sqrt{2})\lambda +(2-\sqrt{2})\gamma \right)/\gamma >0$, there exists one zero at least in $\left(0,\left(2-\sqrt{2}\right)/2\gamma \right)$. The smallest positive zero of *ϕ* in $\left(0,\left(2-\sqrt{2}\right)/2\gamma \right)$ is denoted by $\hat{r}$. Recall that *r* = min⁡{*ρ*, *σ*}; here *ρ* and *σ* are given in (44) and (16), respectively. Let

(46) $$\begin{array}{c} r^{*}=\min\left\{r,\hat{r},\tilde{r}\;\right\}, \end{array}$$

where $\tilde{r}$ is given by

(47) $$\begin{array}{c} \tilde{r}=\frac{\left(4-3\lambda \right)-\sqrt{{\left(4-3\lambda \right)}^{2}-8{\left(1-\lambda \right)}^{2}}}{4\gamma \left(1-\lambda \right)}. \end{array}$$

**Theorem 17**

Suppose that *f*(*ζ*) = 0, *f*′(*ζ*) is full row rank, and *f* satisfies the *γ*-condition (26) on *B*(*ζ*, *r*). Assume that rank⁡*f*′(*x*) ≤ rank⁡*f*′(*ζ*) and that

(48) $$\begin{array}{c} ||\left[I_{{\mathbb{R}}^{n}}-f^{\prime }{\left(x\right)}^{†}f^{\prime }\left(x\right)\right]\left(x-\zeta \right)||\leq \theta {||x-\zeta ||}^{2}, \end{array}$$

for any *x* ∈ *B*(*ζ*, *r*). Let {*x*_*k*_} be sequence generated by inexact Newton-Gauss method with any initial point *x*_0_ ∈ *B*(*ζ*, r*)∖{*ζ*} and the conditions for the control residual *r*_*k*_ and the forcing term *λ*_*k*_:

(49) $$\begin{array}{c} ||f^{\prime }{\left(x_{k}\right)}^{†}r_{k}||\leq {\left(\lambda_{k}||f^{\prime }{\left(x_{k}\right)}^{†}f(x_{k})||\right)}^{2},\\ 0\leq \lambda_{k}\kappa \left(f^{\prime }\left(x_{k}\right)\right)\leq \lambda ,\;k=0,1,2,\ldots . \end{array}$$

Then, {*x*_*k*_} converges to a zero *ζ* of *f*(·) in $\bar{B(\zeta ,r)}$ and *x*_0_ is an approximate zero of *f* in sense of ||*x*_*k*_ − *ζ*||.

One typical and important class of examples satisfying the *γ*-conditions is the one of analytic functions. Following Smale's idea in [2], Shub and Smale introduced in [14] the following invariant for analytic underdetermined systems with surjective *f*′(*ζ*):

(50) $$\begin{array}{c} \gamma \left(f,\zeta \right):=\underset{k>1}{\sup}{||f^{\prime }{\left(\zeta \right)}^{†}\frac{f^{\left(k\right)}(\zeta )}{k!}||}^{1/\left(k-1\right)}. \end{array}$$

For the case when *f*′(*x*) is not surjective, due to loss of the information on im*f*′(*ζ*)^⊥^, Dedieu and Shub introduce in [15] the following invariant for analytic overdetermined systems:

(51) $$\begin{array}{c} \gamma_{1}\left(f,\zeta \right):=\underset{k>1}{\sup}{\left(||f^{\prime }{\left(\zeta \right)}^{†}||\frac{||f^{\left(k\right)}\left(\zeta \right)||}{k!}\right)}^{1/\left(k-1\right)}. \end{array}$$

By [25, Proposition 5.2], one has that an analytic operator satisfies the *γ*-condition and the modified *γ*-condition. So, the conclusions of Theorem 17 still hold when *f* is analytic.

## 4. Proofs

In this section, we prove our main results of local convergence for inexact Newton-Gauss method (7) given in Section 3.

### 4.1. Proof of Theorem 9

The following lemma gives a perturbation bound for Moore-Penrose inverse, which is stated in [31, Corollary 7.1.1 and Corollary 7.1.2].

**Lemma 18 (see [31])**

Let *A* and *B* be *m* × *n* matrices and let *r* ≤ min⁡{*m*, *n*}. Suppose that rank⁡*A* = *r*, 1 ≤ rank⁡*B* ≤ rank⁡*A*, and ||*A* ^†^||||*B* − *A*|| < 1. Then, rank⁡*B* = *r* and

(52) $$\begin{array}{c} ||B^{†}||\leq \frac{||A^{†}||}{1-||A^{†}||||B-A||}. \end{array}$$

**Lemma 19**

Suppose that *f*′ satisfies the modified* L*-average Lipschitz condition on *B*(*ζ*, *r*) and that ||*ζ* − *x*|| < min⁡{*ρ*, *ξ*}, where *r*, *ρ*, and *ξ* are defined in (20), (15), and (14), respectively. Then, rank⁡*f*′(*x*) = rank⁡*f*′(*ζ*) and

(53) $$\begin{array}{c} ||f^{\prime }{\left(x\right)}^{†}||\leq -\frac{||f^{\prime }{\left(\zeta \right)}^{†}||}{h_{0,0}^{\prime }\left(||x-\zeta ||\right)}. \end{array}$$

**Proof**

Since *h*_0,0_′(0) = −1, we have

(54) ||f′(ζ)†||||f′(x)−f′(ζ)|| ≤∫0||x−ζ||L(u)du=h0,0′(||x−ζ||)−h0,0′(0) <−h0,0′(0)=1.

It follows from Lemma 18 that rank⁡*f*′(*x*) = rank⁡*f*′(*ζ*) and

(55) ||f′(x)†||≤||f′(ζ)†||1−(h0,0′(||x−ζ||)−h0,0′(0))=−||f′(ζ)†||h0,0′(||x−ζ||).

**Proof of Theorem 9**

We will prove by induction that {*t*_*k*_} is the majorizing sequence for {*x*_*k*_}; that is,

(56) $$\begin{array}{c} ||\zeta -x_{j}||\leq t_{j}\;\forall j=0,1,2,\ldots . \end{array}$$

Because *t*_0_ = ||*x*_0_ − *ζ*||, thus (56) holds, for *j* = 0. Now, we assume that ||*ζ* − *x*_*j*_|| ≤ *t*_*j*_, for some *j* = *k* ∈ *ℕ*. For the case *j* = *k* + 1, we first notice that

(57) xk+1−ζ =xk−ζ−f′(xk)†[f(xk)−f(ζ)]+f′(xk)†rk =f′(xk)†[f(ζ)−f(xk)−f′(xk)(ζ−xk)]  +f′(xk)†rk  +[Iℝn−f′(xk)†f′(xk)](xk−ζ) =f′(xk)†∫01[f′(xk)−f′(ζ+τ(xk−ζ))]‍(xk−ζ)dτ  +f′(xk)†rk  +[Iℝn−f′(xk)†f′(xk)](xk−ζ).

By using the modified *L*-average Lipschitz condition (25), Lemma 19, the inductive hypothesis (56), and Lemma 1, one has that

(58) ||f′(xk)†∫01‍[f′(xk)−f′(ζ+τ(xk−ζ))](xk−ζ)dτ|| ≤−1h0,0′(||xk−ζ||)  ×∫01||f′(ζ)†||||f′(xk)−f′(ζ+τ(xk−ζ))||  ×||xk−ζ||dτ‍ ≤−1h0,0′(||xk−ζ||)∫01∫τ||xk−ζ||||xk−ζ||L(u)||xk−ζ||du dτ‍‍ =−1h0,0′(||xk−ζ||)  ×∫01hλ,0′(||xk−ζ||)−hλ,0′(τ||xk−ζ||)||xk−ζ||dτ·||xk−ζ||2 ≤−1h0,0′(tk)∫01hλ,0′(tk)−hλ,0′(τtk)tkdτ·||xk−ζ||2‍ =−1h0,0′(tk)(tkh′λ,0(tk)−hλ,0(tk))||xk−ζ||2tk2.

Thanks to (29),

(59) $$\begin{array}{c} ||f^{\prime }{\left(x_{k}\right)}^{†}r_{k}||\leq ||f^{\prime }{\left(x_{k}\right)}^{†}||||r_{k}||\leq \lambda_{k}||f^{\prime }{\left(x_{k}\right)}^{†}||||f\left(x_{k}\right)||. \end{array}$$

Since

(60) −f(xk)=f(ζ)−f(xk)−f′(xk)(ζ−xk) +f′(xk)(ζ−xk)=∫01‍[f′(xk)−f′(ζ+τ(xk−ζ))](xk−ζ)dτ +f′(xk)(ζ−xk),

combining Lemmas 1 and 19, the modified *L*-average Lipschitz condition (25), the inductive hypothesis (56), and the condition (29), we have

(61) λk||f′(xk)†||||f(xk)|| ≤λk||f′(xk)†||∫01||f′(xk)−f′(ζ+τ(xk−ζ))||‍  ×||xk−ζ||dτ  +λk||f′(xk)†||||f′(xk)||||xk−ζ|| ≤−λ(h0,0′(tk))(tkhλ,0′(tk)−hλ,0(tk))||xk−ζ||2tk2  +λtk·||xk−ζ||tk

(62) $$\begin{array}{c} \leq \lambda \frac{\lambda t_{k}+h_{\lambda ,0}\left(t_{k}\right)}{h_{0,0}^{\prime }\left(t_{k}\right)}\cdot \frac{||x_{k}-\zeta ||}{t_{k}}.\;\;\;\;\;\;\;\; \end{array}$$

Combining (28), (58), (59), and (62), we can obtain that

(63) ||xk+1−ζ|| ≤[−tkhλ,0′(tk)−hλ,0(tk)h0,0′(tk)+λ·λtk+hλ,0(tk)h0,0′(tk)+θtk]  ×||xk−ζ||tk =[−tk+(1+λ)(λtkh0,0′(tk)+hλ,0(tk)h0,0′(tk))+θtk]  ×||xk−ζ||tk.

By the definition of *h*_*λ*,0_(*t*), we have *h*_*λ*,0_(*t*)≥−(1 + *λ*)*t*. Then, we can obtain that

(64) $$\begin{array}{c} \left(1+\lambda \right)\left(\frac{\lambda t_{k}}{h_{0,0}^{\prime }\left(t_{k}\right)}+\frac{h_{\lambda ,0}\left(t_{k}\right)}{h_{0,0}^{\prime }\left(t_{k}\right)}\right)\leq \frac{h_{\lambda ,0}\left(t_{k}\right)}{h_{0,0}^{\prime }\left(t_{k}\right)}. \end{array}$$

Note that −1 < *h*_0,0_′(*t*) < 0, for any *t* ∈ (0, *ρ*), and

(65) (1+λ)(λtkh0,0′(tk)+hλ,0(tk)h0,0′(tk))+θtk ≤hλ,0(tk)h0,0′(tk)+θtk≤hλ,0(tk)−θtkh0,0′(tk)=hλ,θ(tk)h0,0′(tk).

Thus, in view of the definition of {*t*_*k*_} given in (21), one has that

(66) $$\begin{array}{c} ||x_{k+1}-\zeta ||\leq \frac{t_{k+1}}{t_{k}}||x_{k}-\zeta ||, \end{array}$$

which implies ||*x*_*k*+1_ − *ζ*|| ≤ *t*_*k*+1_. Therefore, the proof by induction is complete. Since {*t*_*k*_} converges to 0 (by Lemma 4), it follows from (56) that {*x*_*k*_} converges to *ζ* and the estimate (30) holds for all *k* ≥ 0. This completes the proof.

### 4.2. Proof of Theorem 12

**Lemma 20**

Suppose that *f*(*ζ*) = 0, *f*′(*ζ*) is full row rank, and *f*′ satisfies the *L*-average Lipschitz condition (24) on *B*(*ζ*, *r*). Then, for any *x* ∈ *B*(*ζ*, *r*), one has rank⁡*f*′(*x*) = rank⁡*f*′(*ζ*) and

(67) $$\begin{array}{c} ||{\left[I_{{\mathbb{R}}^{n}}-f^{\prime }{\left(\zeta \right)}^{†}\left(f^{\prime }\left(\zeta \right)-f^{\prime }\left(x\right)\right)\right]}^{-1}||\leq -\frac{1}{h_{0,0}^{\prime }\left(||x-\zeta ||\right)}. \end{array}$$

**Proof**

Since *h*_0,0_′(0) = −1, we have

(68) ||f′(ζ)†[f′(x)−f′(ζ)]|| ≤∫0||x−ζ||L(u)du=h0,0′(||x−ζ||)−h0,0′(0) <−h0,0′(0)=1.

It follows from Banach lemma that [*I*_ℝ^*n*^_ − *f*′(*ζ*)^†^(*f*′(*ζ*) − *f*′(*x*))]^−1^ exists and

(69) $$\begin{array}{c} ||{\left[I_{{\mathbb{R}}^{n}}-f^{\prime }{\left(\zeta \right)}^{†}\left(f^{\prime }\left(\zeta \right)-f^{\prime }\left(x\right)\right)\right]}^{-1}||\leq -\frac{1}{h_{0,0}^{\prime }\left(||x-\zeta ||\right)}. \end{array}$$

Since *f*′(*ζ*) is full row rank, we have *f*′(*ζ*)*f*′(*ζ*)^†^ = *I*_ℝ^*m*^_ and

(70) $$\begin{array}{c} f^{\prime }\left(x\right)=f^{\prime }\left(\zeta \right)\left[I_{{\mathbb{R}}^{n}}-f^{\prime }{\left(\zeta \right)}^{†}\left(f^{\prime }\left(\zeta \right)-f^{\prime }\left(x\right)\right)\right], \end{array}$$

which implies that *f*′(*x*) is full row rank; that is, rank⁡*f*′(*x*) = rank⁡*f*′(*ζ*).

**Proof of Theorem 12**

Let $\hat{f}:B(\zeta ,r)\to {\mathbb{R}}^{m}$ be defined by

(71) $$\begin{array}{c} \hat{f}\left(x\right)=f^{\prime }{\left(\zeta \right)}^{†}f\left(x\right),\;x\in B\left(\zeta ,r\right), \end{array}$$

with residual ${\hat{r}}_{k}=f^{\prime }{\left(\zeta \right)}^{†}r_{k}$. Since

(72) $$\begin{array}{c} {\hat{f}}^{\prime }{\left(x\right)}^{†}={\left[f^{\prime }{\left(\zeta \right)}^{†}f^{\prime }\left(x\right)\right]}^{†}=f^{\prime }{\left(x\right)}^{†}f^{\prime }\left(\zeta \right),\;x\in B\left(\zeta ,r\right), \end{array}$$

one has that {*x*_*k*_} coincides with the sequence generated by inexact Newton-Gauss method (7) for $\hat{f}$. In addition, we have

(73) $$\begin{array}{c} {\hat{f}}^{\prime }{\left(\zeta \right)}^{†}={\left(f^{\prime }{\left(\zeta \right)}^{†}f^{\prime }\left(\zeta \right)\right)}^{†}=f^{\prime }{\left(\zeta \right)}^{†}f^{\prime }\left(\zeta \right), \end{array}$$

and so

(74) $$\begin{array}{c} ||{\hat{f}}^{\prime }{\left(\zeta \right)}^{†}\hat{f}\left(\zeta \right)||=||f^{\prime }{\left(\zeta \right)}^{†}f^{\prime }\left(\zeta \right)f^{\prime }{\left(\zeta \right)}^{†}f\left(\zeta \right)||=||f^{\prime }{\left(\zeta \right)}^{†}f\left(\zeta \right)||. \end{array}$$

Because ||*f*′(*ζ*)^†^ *f*(*ζ*)|| = ||Π_*ker*⁡*f*′(*ζ*)^⊥^_|| = 1, thus, we have $||{\hat{f}}^{\prime }{\left(\zeta \right)}^{†}||=||{\hat{f}}^{\prime }{\left(\zeta \right)}^{†}\hat{f}(\zeta )||=1$. Therefore, by (24), we can obtain that

(75) ||f^′(ζ)†||||f^′(x)−f^′(ζ+τ(x−ζ))|| =||f′(ζ)†(f′(x)−f′(ζ+τ(x−ζ)))|| ≤∫τ||x−ζ||||x−ζ||L(u)du.

That is, $\hat{f}$ satisfies the modified *L*-average Lipschitz condition (25) on *B*(*ζ*, *r*). So, Theorem 9 is applicable and {*x*_*k*_} converges to *ζ* as follows. Note that ${\hat{f}}^{\prime }{\left(\cdot \right)}^{†}\hat{f}(\cdot )=f^{\prime }{\left(\cdot \right)}^{†}f(\cdot )$ and *f*(·) = *f*′(·)*f*′(·)^†^ *f*(·); it follows that *ζ* is a zero of *f*.

### 4.3. Proof of Theorem 13

**Lemma 21**

Suppose that *f*(*ζ*) = 0, *f*′(*ζ*) is full row rank, and *f*′ satisfies the* L*-average Lipschitz condition (24) on *B*(*ζ*, *r*). Then, one has

(76) $$\begin{array}{c} ||f^{\prime }{\left(x\right)}^{†}f^{\prime }\left(\zeta \right)||\leq -\frac{1}{h_{0,0}^{\prime }\left(||x-\zeta ||\right)},\;x\in B\left(\zeta ,r\right). \end{array}$$

**Proof**

Since *f*′(*ζ*) is full row rank, we have *f*′(*ζ*)*f*′(*ζ*)^†^ = *I*_ℝ^*m*^_. It follows that

(77) f′(x)†f′(ζ)(Iℝn−f′(ζ)†(f′(ζ)−f′(x))) =f′(x)†f′(x), x∈B(ζ,r).

By Lemma 20, *I*_ℝ^*n*^_ − *f*′(*ζ*)^†^(*f*′(*ζ*) − *f*′(*x*)) is invertible for any *x* ∈ *B*(*ζ*, *r*). Thus, in view of the equality *A* ^†^ *A* = Π_*ker*⁡*A*^⊥^_, for any *m* × *n* matrix *A*, one has that

(78) f′(x)†f′(ζ) =Πker⁡f′(x)⊥[Iℝn−f′(ζ)†(f′(ζ)−f′(x))]−1.

Therefore, Lemma 20 is applicable to conclude that

(79) ||f′(x)†f′(ζ)|| ≤||Πker⁡f′(x)⊥||||[Iℝn−f′(ζ)†(f′(ζ)−f′(x))]−1|| ≤−1h0,0′(||x−ζ||).

**Proof of Theorem 13**

Using Lemma 21, *L*-average condition (24), and the residual condition (35), respectively, instead of Lemma 19, modified *L*-average condition (25), and condition (29), one can complete the proof of Theorem 13 as the same line of proof in Theorem 9.

### 4.4. Proof of Theorem 17

**Proof**

Recall that the majorizing sequence {*t*_*k*_} is defined by (21) and the majorizing function *h*_*λ*,*θ*_(*t*) is defined by (42). By Lemma 4, {*t*_*k*_} is strictly decreasing and converges to 0. We first note that (57) gives

(80) xk−ζ=f′(xk)†∫01‍[f′(xk)−f′(ζ+τ(xk−ζ))]       ×(xk−ζ)dτ+f′(xk)†rk +[Iℝn−f′(xk)†f′(xk)](xk−ζ).

Using Lemma 8, the *γ*-condition (26), and Lemma 21, one has that

(81) ||f′(xk)†∫01[f′(xk)−f′(ζ+τ(xk−ζ))](xk−ζ)dτ|| ≤γtk21−4γtk+2γ2tk2·||xk−ζ||tk2.

Thanks to (60), we use the *γ*-condition (26), Lemma 21, and the condition (49) to obtain that

(82) λk||f′(xk)†f(xk)|| ≤λ(2γ2tk2−3γtk+1)tk2γ2tk2−4γtk+1·||xk−ζ||tk.

Note that

(83) λ(1−3γt+2γ2t2)1−4γt+2γ2t2<1for  any  0≤t<(4−3λ)−(4−3λ)2−8(1−λ)24γ(1−λ).

Then, it follows from (49) and (82) that

(84) ||f′(xk)†rk||≤(λk||f′(xk)†f(xk)||)2≤λ(2γ2tk2−3γtk+1)tk22γ2tk2−4γtk+1·||xk−ζ||2tk2.

Thus, we can obtain by combining (81), (84), and (48) that

(85) ||xk+1−ζ|| ≤(γtk21−4γtk+2γ2tk2+λ(2γ2tk2−3γtk+1)tk21−4γtk+2γ2tk2+θtk2)  ×||xk−ζ||2tk2.

It is clear that the function

(86) $$\begin{array}{c} \psi \left(t\right):=\frac{\lambda \left(1-3\gamma t+2\gamma^{2}t^{2}\right)}{1-4\gamma t+2\gamma^{2}t^{2}}+\frac{\gamma }{1-4\gamma t+2\gamma^{2}t^{2}}+\theta \end{array}$$

is increasing monotonically with respect to *t* in $\left[0,\left(2-\sqrt{2}\right)/2\gamma \right)$. Hence, we have

(87) ||xk−ζ|| ≤(γt021−4γt0+2γ2t02+λ(2γ2t02−3γt0+1)t021−4γt0+2γ2t02+θt02)  ×||xk−1−ζ||2t02 ≤(ψ(t0)t0)2k−1||x0−ζ||.

Consequently, to show that *x*_0_ is an approximate zero of *f*, it suffices to prove *ψ*(*t*_0_)*t*_0_ ≤ 1/2. In fact, in view of the definition of r* given in (46), for any $t\in [0,\hat{t}\;]$, we have −1 ≤ *ϕ*(*t*) ≤ 0. Consequently, we get that

(88) $$\begin{array}{c} \frac{4\gamma^{2}\left(\lambda +\theta \right)t^{3}-2\gamma \left(3\lambda +4\theta \right)t^{2}+2\left(\lambda +\theta +\gamma \right)t}{1-4\gamma t+2\gamma^{2}t^{2}}\leq 1, \end{array}$$

which is equivalent to $t\psi (t)\leq 1/2,t\in [0,\hat{t}\;]$. The proof is complete.
